# Comparative analysis of supervised learning models for effluent quality prediction in wastewater treatment plants

**DOI:** 10.1371/journal.pone.0325234

**Published:** 2025-06-10

**Authors:** Liu Bo-qi, Zhou Ding-jie, Zhao Yang, Shi Long-yu

**Affiliations:** 1 State Key Laboratory of Regional and Urban Ecology, Institute of Urban Environment, Chinese Academy of Sciences, Xiamen, Fujian, China; 2 Rural Revitalization College, Fujian Agriculture and Forestry University, Fuzhou, China; SUMAIT University, TANZANIA, UNITED REPUBLIC OF

## Abstract

Effluent quality prediction is critical for optimizing Wastewater Treatment Plant (WWTP) operations, ensuring regulatory compliance, and promoting environmental sustainability. This study evaluates the performance of five supervised learning models—AdaBoost, Backpropagation Neural Networks (BP-NN), Support Vector Machine (SVR), XGBoost, and Gradient Boosting (GB)—using data from a WWTP in Zhuhai, China. The Effluent Quality Index (EQI), integrating multiple pollutant concentrations and environmental impacts, was used as the target variable. The models were trained and tested on 84 monthly datasets, with their performances compared using R^2^, Mean Absolute Percentage Error (MAPE), and Mean Bias Error (MBE). XGBoost achieved the best balance between accuracy and robustness, with the lowest MAPE(6.11%) and a high R^2^(0.813), while SVR excelled in fitting accuracy(R^2^ = 0.826) but showed limitations in error control. Although we employed GridSearchCV with cross-validation to optimize hyperparameters and ensure a fair model comparison, the study is limited by the reliance on data from a single WWTP and the relatively small dataset size (84 records). Nevertheless, the findings provide valuable insights into selecting effective machine learning models for effluent quality prediction, supporting data-driven decision-making in wastewater management and advancing intelligent process optimization in WWTP.

## 1 Introduction

Wastewater treatment is a key topic in modern environmental protection. As urbanization and industrialization accelerate, water pollution has become more severe. Therefore, effectively controlling and assessing effluent water quality is essential for the optimal operation of wastewater treatment systems. Accurate prediction of effluent water quality optimizes treatment processes, enhances energy efficiency, and reduces operational uncertainty. It also supports the sustainable development of ecological systems [1–4].

In recent years, machine learning has made significant progress in predicting complex environmental systems, demonstrating powerful data-driven analytics in a variety of fields. For example, in the field of financial prediction, Convolutional Neural Networks (CNN) have been used in Stock Price Prediction [5], while Deep Learning has been successfully applied to Cross-Market Arbitrage [6], improving the ability to recognize market dynamics and the accuracy of trading strategies. In the field of energy and geoscience engineering, machine learning has also been widely applied to the prediction and optimization of key parameters, such as the prediction of sand production in gas wells [7] and the prediction of bubble pressure in oil reservoirs [8]. These studies demonstrate that intelligent prediction models can capture complex data patterns and support system regulation. They also show strong adaptability in nonlinear and dynamic environments.

Traditional regulation methods, such as empirical or univariate models, often struggle with the nonlinear and multivariate nature of wastewater systems. As a result, their prediction accuracy is low, particularly when pollutant concentrations vary significantly [9]. In contrast, in recent years, supervised learning-based machine learning methods have demonstrated strong adaptability and efficient prediction capabilities when dealing with nonlinear and complex pollutant data [10–12].

Existing studies have shown significant progress in the application of supervised learning models in wastewater treatment. For example, Jafar R et al. successfully predicted key pollutants such as Chemical Oxygen Demand (COD) and Biochemical Oxygen Demand (BOD) in a wastewater treatment system using artificial neural networks (ANN) [13].Xie Y et al. used feed-forward neural networks (FFNN) combined with genetic algorithms to significantly improve the prediction accuracy of COD and Total Phosphorus (TN) [14]. In addition, Hamada M S et al. compared a variety of supervised learning models, such as random forest and Gaussian process regression models, and demonstrated their superior performance in water quality prediction [6].

However, these single metrics, while able to reflect the levels of specific pollutants, cannot comprehensively measure the overall impact of wastewater treatment on the environment and ecosystems. To address this issue, this paper introduces the Effluent Quality Index (EQI), which integrates the concentrations of multiple pollutants and their potential harmfulness to the environment, and is able to comprehensively reflect the impact of wastewater treatment on the environment [15]. In order to more accurately capture the complex relationship of multiple pollutants in effluent water quality and to improve the comprehensiveness and accuracy of prediction, this paper further introduces a variety of supervised learning models for comparative study. Models such as AdaBoost, BP-NN, SVR, XGBoost, and GB are effective in handling complex, nonlinear, and multivariate systems. Their versatility allows for robust comparisons and reliable predictions across diverse features and noise levels.

The Adaboost model is an integrated learning algorithm that improves the overall prediction accuracy by iterating the weak learner multiple times. It is particularly suitable for dealing with noisy and unbalanced samples.The Adaboost model improves the accuracy of the model by adjusting the weights of misclassified samples in each iteration [16]. In wastewater treatment studies, Adaboost models can be used to predict the concentration of key pollutants. For example, Huixin Chen et al predicted EQI of a wastewater treatment plant using five different models, of which the Adaboost model performed the most prominently on the test set, with the lowest RMSE and MAE of all models, and demonstrated strong generalisation on the test set [17]. Therefore, the Adaboost model was selected for this study to explore the volatility and uncertainty in effluent water quality data.

BP-NN is a common multi-layer feed-forward neural network capable of optimising weights through back propagation algorithms and is one of the foundations of modern deep learning [18]. BP neural networks are commonly used to solve nonlinear problems such as prediction tasks in multivariate systems.Lin W et al. implemented BP neural networks to predict COD values in wastewater treatment processes.Their study confirmed that BP neural networks can capture the nonlinear relationship between influent factors and effluent COD values, thus allowing operators to adjust reactor conditions to improve performance [19]. Therefore, the BP neural network was chosen to handle the complex interactions between pollutants in this study.

On the other hand, SVR is able to handle complex nonlinear regression tasks by mapping data into a high-dimensional space through kernel functions, and are particularly suitable for prediction tasks with small samples or high-dimensional data [20]. Liu Z et al. successfully applied an SVR model to predict the concentration of dissolved oxygen (DO) in an anoxic river system, and the study demonstrated that SVR can effectively simulate temperature, pH and flow rate and other non-linear relationships between input variables, thus improving the prediction accuracy of complex water treatment systems [21]. Sahu et al. integrated SVR with wavelet transform and principal component analysis (PCA) to BOD in aqueous systems. It was found that SVR performed well in reducing prediction errors and handling non-linear datasets [22]. Granata et al. demonstrated that SVR exhibited superior performance compared to RT in predicting TSS, TDS, and COD, while both models achieved comparable accuracy in estimating BOD5 [23]. Therefore, SVR was chosen in this study to deal with complex nonlinear and multidimensional features in wastewater treatment data.

XGBoost is a widely used integrated learning algorithm with efficient computational performance and powerful processing capability, while its regularisation mechanism can effectively prevent model overfitting [24,25]. Gradient boosting (GB) is also a tree-based integrated learning algorithm, as is XGBoost, and they are able to effectively deal with noisy, missing-value-containing data by constructing multiple weak decision trees and progressively improving the fit of the model [26].The study by Khan et al. used GB and XGBoost models to predict water quality indices. The study showed that these models have the ability to handle partial or incomplete datasets and are suitable for real-time water quality monitoring due to their strong predictive power and performance in coping with noisy data [27]. Panda P et al. applied gradient boosting technique for water quality prediction, showing how the GB model can efficiently deal with missing and noisy by iteratively minimising the error by using weak decision tree data [28].

Although various supervised learning models have been successfully applied in wastewater treatment, most existing studies have focused on predicting individual water quality indicators such as TN, TP, COD, BOD, NH_3_-N, and NH_4_^+^ [29–32],with limited attention given to comprehensive indices like EQI, which integrates multiple parameters into a single metric for a more holistic assessment of water quality. This study fills this gap by evaluating five widely used supervised learning models—AdaBoost, BP-NN, SVR, XGBoost, GB—within the same experimental framework, ensuring an objective and fair assessment of their predictive effectiveness and stability in EQI prediction.

Beyond methodological contributions, the findings of this study also have practical implications for WWTP, providing valuable guidance on selecting the most suitable machine learning model for effluent quality prediction. By improving the accuracy and reliability of effluent predictions, this study contributes to the development of intelligent decision-making systems, facilitating process optimization and enhancing wastewater management efficiency.

The selection of these five models was based on their algorithmic diversity and proven applicability in environmental modeling tasks with structured, small-to-medium-sized datasets. Ensemble methods (AdaBoost and GB) offer robustness against overfitting and are effective in capturing nonlinear interactions. Support vector regression (SVR) excels in high-dimensional and small-sample scenarios due to its margin-based optimization. BP neural networks provide flexible function approximation capabilities, while XGBoost, as a regularized boosting technique, balances prediction accuracy and computational efficiency.Although other models such as Random Forest, K-Nearest Neighbors (KNN), and LSTM have been successfully applied in related studies, they were not included in this work due to specific limitations. Random Forest has been extensively studied in similar contexts. KNN, while simple, often lacks robustness and interpretability in structured regression tasks. LSTM requires longer and denser time-series data than the monthly records available in this study. The final model selection reflects a balance between algorithmic diversity, efficiency, and suitability for the current dataset. The final model selection was guided by considerations of algorithmic diversity, interpretability, computational efficiency, and compatibility with the characteristics of the available dataset.

This study aimed to systematically compare multiple supervised learning models to identify the most effective approach for effluent quality prediction. By analyzing their performance under a unified experimental framework, we provided an objective evaluation that can guide wastewater treatment plants in selecting optimal machine learning models for improved process control and environmental compliance. To facilitate clarity and consistency, the key abbreviations used throughout this study are summarized in Table 1.

**Table 1:** Abbreviation Table.

Abbreviation Table	Full Term
WWTP	Wastewater Treatment Plant
AdaBoost	Adaptive Boosting
BP-NN	Back propagation neural networks
SVR	Support Vector Regression
XGBoost	eXtreme Gradient Boosting
GB	Gradient boosting
EQI	Effluent Quality Index
BOD	Biochemical Oxygen Demand
COD	Chemical Oxygen Demand
SS	Suspended Solids
TP	Total Phosphorus
TN	Total Nitrogen

## 2 Data and methods

To ensure the accuracy and robustness of effluent quality prediction, this study follows a structured methodology that encompasses data collection, preprocessing, model selection, and evaluation. In this part, we introduce the dataset used and research framework in this research, detailing its source, characteristics, and key variables. Next, we outline the data preprocessing steps, including normalization techniques and dataset partitioning, to ensure unbiased and reliable model training. Following this, we describe the principles and implementation of five supervised learning models—AdaBoost, BP-NN,SVR,XGBoost, and GB)—highlighting their theoretical foundations and relevance to wastewater quality prediction. Finally, we present the evaluation metrics and hyperparameter tuning strategies employed to optimize model performance, ensuring a comprehensive and objective comparison of different machine learning techniques.

### 2.1 Data sources and research framework

The research data of this study comes from the actual operation data of a wastewater treatment plant in Guangdong Province, China. The existing project scale is 50,000 m^3^/d, which was put into operation in January 2006, with a daily treatment scale of 50,000 m³/d, and the main process of which is Aubert oxidation ditch and spoke-flow type The main process is Aubert oxidation ditch and radial flow type secondary sedimentation tank, and the effluent water quality is in line with Grade A standard of Pollutant Emission Standard for Urban Sewage Treatment Plant (GB 18918−2002). Within the service area, the pipeline network is arranged by rainwater and sewage diversion, and lifting pumping stations are set up along the way for transmission. Among them, the sewage trunk pipe is laid with DN1000-DN1500 pipes, and the sewage collection rate reaches 90%; during the rainy season, the surface aggregated runoff is discharged into the rainwater pipe network, and finally enters the plant. The data time used in this paper is the whole year from 2014 to 2020, removing some abnormal null values, there are 84 monthly data. The main data are BOD, COD, SS, TP, TN in and out of the water content, the cumulative amount of treated water and cumulative electricity consumption.BOD, COD, SS, TP, TN all in (mg/L).

This study follows a structured methodology to ensure the reliability and fairness of the model comparison. First, we construct a comprehensive dataset based on real-world WWTP records. Second, we select five supervised learning models based on their proven effectiveness in handling nonlinear relationships, high-dimensional data, and small datasets. Third, we introduce the EQI as an integrated metric to better capture the overall environmental impact of effluent discharge. Finally, we optimize the hyperparameters of each model using GridSearchCV to ensure robust and unbiased performance evaluation.

### 2.2 Data pre-processing

Due to the large scale differences in the data, direct modelling using raw data may affect the convergence speed and prediction accuracy of the model. Therefore, we normalised the data before it was input into the model. The normalisation process uses the Min-Max normalisation method, which scales the data to a range of 0–1 to eliminate scale differences between different features. The mathematical expressions are as follows:


$$X_{scaled}=\frac{X-X_{\min}}{X_{\max}-X_{\min}}$$ (1)

whereX denotes the feature matrix and $X_{\max}$ and $X_{\min}$ are the minimum and maximum values in the feature matrix, respectively, and $X_{scaled}$ is the normalised feature matrix.

The dataset of this study consists of monthly data from 84 WWTP. To ensure the generalisation ability of the model and the accuracy of the evaluation, the dataset was processed using random upsetting to prevent the data order from biasing the model training. By randomly disrupting the index, the dataset was divided into two parts: the first 50 of these samples were used as the training set, and the remaining 34 were used as the test set. Such a division ensures that the data distributions of the training and test sets are as close as possible to the overall distribution of the data, which helps to obtain more stable model evaluation results.

#### 2.2.1 Calculation of EQI.

In order to evaluate effluent quality more comprehensively, this study introduces EQI, which provides an integrated measure of water quality after treatment. Unlike conventional single-indicator evaluations that focus on individual pollutant concentrations such as BOD, COD, and TN, EQI is designed to reflect the overall environmental impact of wastewater discharge by incorporating multiple key pollutants and their relative importance. Existing approaches to wastewater assessment often rely on specific pollutant thresholds to determine compliance with discharge regulations. However, these approaches fail to capture the combined effect of multiple pollutants, which interact in complex ways to influence water quality and ecosystem health. Moreover, traditional single-indicator methods do not account for the relative significance of different pollutants, treating all pollutants as independent factors rather than considering their cumulative environmental impact. To address these limitations, EQI integrates multiple pollutants into a single index, offering a more holistic evaluation of effluent quality.Previous studies have applied EQI in effluent assessment and demonstrated its effectiveness in capturing overall treatment performance under varying conditions [33–35].

The EQI is computed using a weighted sum of key pollutant concentrations, allowing for a comprehensive assessment of wastewater treatment performance. The calculation formula is given as:


$$EQI=\sum_{d=1}^{5}W_{d}\times Index_{d}$$ (2)

where: $Index_{d}$ indicates the effluent concentration of BOD5, COD, TN, TP, SS pollutants, mg/L; $W_{d}$ indicates the weight, COD as the reference pollutant, the weight is the ratio of the effluent concentration of each pollutant to the effluent concentration of COD.

EQI provides a more comprehensive assessment of water quality compared to individual pollutant indicators, as it considers the combined impact of multiple pollutants rather than evaluating them in isolation. The integration of pollutant weighting allows better differentiation between effluent samples, making it useful for wastewater treatment plant optimization and regulatory decision-making. While EQI offers a unified and adaptable approach for assessing effluent quality, further validation is necessary to confirm its effectiveness in capturing overall treatment performance. Future research should compare EQI with other composite water quality indices and assess its applicability across multiple treatment facilities to ensure its robustness and reliability.

### 2.3 Model principles

#### 2.3.1 Principle of the AdaBoost regression model.

The AdaBoost regression model enhances prediction accuracy by integrating multiple weak learners, which in this study are decision tree regressors, into a strong learner. During each iteration, the AdaBoost model improves overall prediction capability by adjusting the weights of samples with larger errors.

Process:

(1) Weak Learner Selection

Decision tree regressors were chosen as weak learners. As a non-parametric model, decision trees partition the feature space to establish segmented predictive functions. The tree depth was limited to 3 to avoid overfitting. The basic structure of decision trees can be represented as follows:


$$h(x)=\sum_{i=1}^{N}w_{i}I(x\in R_{i})$$ (3)

where, $R_{i}$ is the partition of the feature space, and$w_{i}$ is the prediction value in partition.

(2) AdaBoost iteratively trains a series of weak learners by adjusting sample weights based on the prediction error from the prior iteration. Each weak learner’s predictions are weighted according to its error, and the final strong learner combines the weighted predictions of all weak learners. The mathematical expression is given as:

$$f(x)=\sum_{m=1}^{M}\alpha_{m}h_{m}(x)$$ (4)

where$\alpha_{m}$ represents the weight of the m-th weak learner, $h_{m}(x)$ is the m-th weak learner, andM is the total number of learners.

(3) Error Update Mechanism:

During each iteration, the model updates sample weights based on prediction errors from the previous round.

#### 2.3.2 Principle of BP neural network regression model.

A BP neural network is a common multi-layer feed-forward neural network that uses an error back propagation algorithm to adjust the network weights for nonlinear regression prediction. In this study, a multilayer perceptron (MLP) is used as a BP neural network model. It contains an input layer, several hidden layers and an output layer. The input layer receives the features of the input data, assuming that the input data has n features, then the input layer contains n neurons. The hidden layer transforms the linear transformation into a nonlinear mapping through an activation function in order to capture the complex relationships in the data. Among the activation functions of the hidden layer, the options are ReLU (Rectified Linear Unit) or tanh (hyperbolic tangent function). The last layer is the output layer, whose number of neurons depends on the number of target variables to be predicted. In regression problems, the output layer usually has only one neuron, which is used to predict the target value. The output of the network is iteratively updated by an error back propagation algorithm.The output of the MLP can be expressed as:


$$T=f(W^{T}X+b)$$ (5)

where W is the weight matrix, X is the input feature matrix, b is the bias term, andf is the activation function.

For optimization, adam (adaptive moment estimation) or lbfgs (a quasi-Newton optimization method) was used. Adam, with its noise-resistant efficiency, is suited for most neural network applications, while lbfgs is more appropriate for small datasets due to its rapid convergence.

#### 2.3.3 Principle of SVR.

SVR is a regression model. Its objective is to identify a function f(P)f(P)f(P) that minimizes the error between predicted outputs (TTT) and actual values, while maintaining model complexity under a specified tolerance. SVR leverages kernel functions to map data into high-dimensional feature space, facilitating the discovery of an optimal linear function for prediction. The general formulation is:


$$f(P)=\sum_{i=1}^{n}\left(\alpha_{i}-\alpha_{i}^{*}\right)K(P_{i},P)+b$$ (6)

where $\alpha_{i}$ and $\alpha_{i}^{*}$ are Lagrange multipliers, $K(P_{i},P)$ is (In this paper, the radial basis kernel function RBF is used, and the equation is $K(P_{i},P_{j})=\exp(-\gamma {\left\|P_{i}-P_{j}\right\|}^{2})$), b is the bias term, $P_{i}$ is the support vector。

SVR performance depends heavily on two hyperparameters: the penalty parameter(C) and kernel parameter(gamma).The penalty parameter(C)controls the tolerance of the model to error and gamma determines the width of the distribution of the RBF kernel function.

#### 2.3.4 Principle of XGBoost.

XGBoost is an optimized implementation of gradient boosting decision trees (GBDT). By iteratively building trees, the model minimizes residuals from previous iterations, progressively improving predictive accuracy. The output at each iteration is expressed as:


$$\overset{\wedge }{\underset{i}{T}}=f_{1}(P_{i})+f_{2}(P_{i})+\cdots f_{K}(P_{i})$$ (7)

where $\overset{\wedge }{T_{i}}$ is the predicted value of the ith sample, $P_{i}$ is the feature of the ith sample, $f_{K}$ is the kth decision tree and is the total number of trees and K is the total number of trees.

XGBoost tunes the model parameters by minimising an objective function that contains a loss function and a regularisation term to balance the complexity of the model with the prediction error. For regression tasks, the squared error is typically used as the loss function:


$$L\left(\theta \right)=\sum_{i=1}^{n}{\left(T_{i}-{\overset{\wedge }{T}}_{i}\right)}^{2}+\sum_{k=1}^{K}\Omega (f_{k})$$ (8)

where $T_{i}$ is the true value, $\overset{\wedge }{\underset{i}{T}}$ is the predicted value, $\Omega (f_{k})$ is the regularisation term to control the model complexity.XGBoost has several important hyperparameters, including the number of trees (n_estimators), learning_rate, tree depth (max_depth), and subsampling ratio (subsample).

#### 2.3.5 Principle of gradient boosting.

GB is an integrated learning algorithm that reduces prediction error by incrementally constructing multiple weak learners, usually decision trees.The core idea of GB is to incrementally improve the model performance by constructing a new decision tree model with each round of iterations that predicts the residuals of the current model.The prediction model of GB can be represented as:


$$\overset{\wedge }{\underset{i}{T}}=\sum_{m=1}^{M}\alpha_{m}h_{m}(P_{i})$$ (9)

where $\overset{\wedge }{\underset{i}{T}}$ is the predicted value of the ith sample, M is the total number of decision trees, $h_{m}$ is the mth decision tree model, $\alpha_{m}$ is the weight (learning rate) of each tree, $P_{i}$ is the input features of the ith sample.

GB updates the model by minimising the squared error of the residuals. For each round of iteration, the model calculates the current residuals and fits a new decision tree to reduce the residuals.The performance of GB is highly dependent on the setting of hyperparameters, such as the number of trees (n_estimators), the depth of the tree (max_depth), and the learning rate (learning_rate).

### 2.4 Indicators for model testing

In this study, the predictive performance of different machine learning models was evaluated using the following statistical metrics:

(1) R^2^ (Coefficient of Determination): R^2^ measures the goodness-of-fit of the model, with values ranging from 0 to 1. A higher R^2^ value indicates that the model explains more variance in the observed data, signifying better predictive accuracy.
(2) MBE (Mean Bias Error): MBE represents the average bias in model predictions by quantifying the difference between predicted and actual values. A lower absolute MBE value indicates a smaller systematic bias, reflecting better predictive reliability.
(3) MAPE (Mean Absolute Percentage Error): MAPE measures the percentage deviation of predicted values from actual values, providing an intuitive measure of prediction accuracy. A lower MAPE indicates a better-performing model with reduced relative error.
(4) MAPE Std Dev (Standard Deviation of MAPE): This metric reflects the variability in MAPE across different test samples. A lower standard deviation suggests that the model produces more consistent predictions across different observations.
(5) 95% CI for MAPE (%): The 95% confidence interval (CI) for MAPE quantifies the range within which the true MAPE value is expected to fall, offering an assessment of prediction uncertainty. A narrower confidence interval indicates higher precision in the model’s predictions.
(6) Paired T-test p-value: The paired T-test is used to determine whether there is a statistically significant difference between predicted and actual values. A p-value greater than 0.05 suggests that the model’s predictions are not significantly different from the observed values, indicating reliable performance.
(7) Wilcoxon test p-value: The Wilcoxon signed-rank test is a non-parametric alternative to the paired T-test, assessing whether the distributions of predicted and actual values significantly differ. A p-value greater than 0.05 further supports that the model’s predictions align well with the actual data.

### 2.5 GridSearchCV parameter settings

In this study, we employed GridSearchCV with 5-fold cross-validation to optimize the hyperparameter settings of each supervised learning model. The hyperparameter selection was guided by a combination of empirical best practices, insights from previous studies, and considerations of computational efficiency. Given the complexity and multivariate nature of wastewater treatment data, our approach aimed to strike a balance between model accuracy, generalization capability, and training efficiency. By systematically searching across predefined parameter grids and evaluating model performance using 5-fold cross-validation and negative mean squared error (neg_mean_squared_error) as the scoring metric, we were able to identify the optimal parameter configurations that enhanced model robustness and predictive accuracy. The detailed grid search settings for each model are presented in Table 2.

**Table 2:** GridSearchCV parameter setting values.

Supervised learning model	GridSearchCV parameter settings
Adaboost	Number of weak learners (n_estimators): list(range(10, 200, 5))
	learning_rate: 0.01, 0.05,0.1 and 0.2
BP neural network	hidden_layer_sizes: set to (50,), (100,), and (50, 50)
	activation function: values of relu and tanh
	Optimisation algorithm (solver): choose adam and lbfgs
	learning_rate_init: set to 0.001, 0.01 and 0.05
	Maximum number of iterations (max_iter): take the value of 1000 and 2000
SVR	Penalty coefficient (C): take the value of 0,2 and 10
	Kernel function parameter (gamma): take the value of −1,1 and 10
	Kernel function (kernel): use RBF (radial basis function)
XGboost	Number of trees (n_estimators): set to 50, 100 and 150
	learning rate (learning_rate): take the value of 0.01, 0.05 and 0.1
	Maximum depth (max_depth): set to 3, 4 and 5
	subsample rate (subsample): take the value of 0.8 and 1.0
GB	number of trees (n_estimators): set to 100 and 150
	Learning rate (learning_rate): values of 0.05 and 0.1
	Maximum depth (max_depth): set to 2 and 3
	min_samples_split: values 2 and 4
	Minimum leaf node samples (min_samples_leaf): set to 1 and 2
	subsample rate (subsample): fixed at 0.8

## 3 Model construction and prediction results

### 3.1 Model construction

Referring to the results of previous studies, this paper uses the influent levels of BOD, COD, SS, TP, and TN, the cumulative treated water volume, and the cumulative electricity consumption as the input features of the prediction model. Before model training, the data were normalized using the Min-Max normalization method, as described in Equation (1), to eliminate scale differences between different features and improve model convergence. EQI, which integrates multiple pollutant concentrations and their environmental impact, was calculated following Equation (2) and used as the target variable.

The dataset consists of 84 months of records from 2014 to 2020, with the first 50 months used as the training set and the remaining 34 months as the test set. Five machine learning models were constructed using Python’s machine learning library. To ensure optimal performance, hyper-parameters were tuned using GridSearchCV, which systematically searches for the best combination of hyper-parameters to improve model accuracy.

### 3.2 Prediction results and analysis

**Fig 1:**
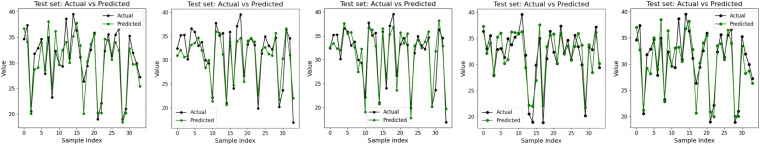
Adaboost 、BP-NN、SVR、XGBoost and GB Comparison of model actual values and test values.

**Fig 2:**
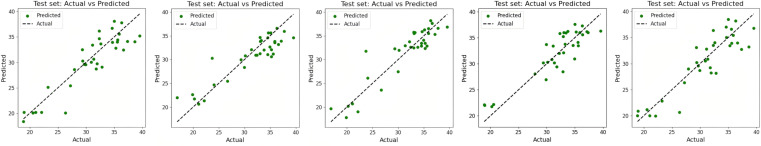
Adaboost 、BP-NN、SVR、XGBoost and GB model fitting results.

**Fig 3:**
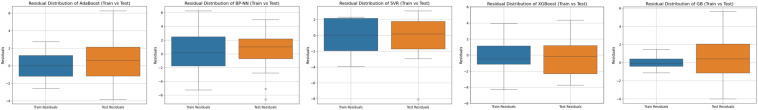
Residual Distribution of Adaboost, BP-NN, SVR, XGBoost, and GB Models.

**Fig 4:**
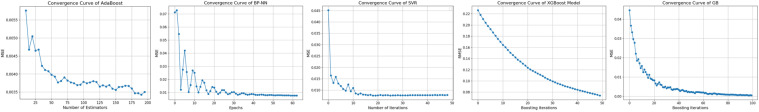
Convergence Curves of Adaboost, BP-NN, SVR, XGBoost, and GB Models.

The test set was predicted using the five trained machine learning models, and Fig 1 shows the comparison between the actual and predicted values of the five models on the test set. Fig 2 shows the comparison of the fitting results for the 5 models, and Table 3 shows the comparison of the test metrics for the 5 models. Fig 3 illustrates the residual distribution of AdaBoost, BP-NN, SVR, XGBoost, and GB models, providing insight into their prediction errors and variability. Fig 4 displays the convergence curves of these models, demonstrating the optimization process and stability of the learning algorithms over iterations.

**Table 3:** Comparison of supervised learning model predictors.

	Adaboost	BP-NN	SVR	XGboost	GB	Linear Regression
R^2^	0.7979	0.8030	0.8382	0.8131	0.7916	0.7358
MBE	−0.6268	−0.6542	0.1124	0.2459	−0.4725	−0.3811
MAPE	6.5528%	6.9562%	6.5775%	6.1108%	6.6048%	8.6149%
MAPE Std Dev	0.7721	1.1719	1.0624	0.7830	0.7976	0.9831
95% CI for MAPE (%)	(5.1233,8.1260)	(4.9020,9.6271)	(4.8061, 8.8652)	(4.6054, 7.6602)	(5.1595, 8.2873)	(6.8429,10.6754)
Paired T-test p-value	0.1480	0.1441	0.7852	0.5236	0.2864	0.4470
Wilcoxon test p-value	0.2214	0.0669	0.9328	0.4989	0.4167	0.4671
Training time	10.18(seconds)	5.04(seconds)	1.27(seconds)	2.84(seconds)	1.97(seconds)	0.01(seconds)

#### 3.2.1 Model prediction and fitting performance.

As can be seen in Fig 1, the predicted trends of all five models largely follow the fluctuations of the actual values. At several locations, such as the high and low points, the predicted values performed better in correspondence with the actual values, indicating that the models were able to capture the trend of the data in the test set to some extent. The predictive ability of all models may be relatively weak at the boundary points of low and high values, with large deviations at some locations.

As can be seen from Fig 2, most of the data points in the scatterplot are relatively concentrated around the ideal prediction line (black dashed line), which indicates that the five models perform better in fitting the test set and predict the values of most samples relatively well. In the intermediate value range (25–35), the performance of most of the models is relatively stable, with the scatter denser and concentrated around the dotted line. This suggests that the models are predicting values that are closer to the actual values when dealing with the middle range of values. In the low (20–25) and high (35–40) value ranges, the performance of some models fluctuates more and the scatter is far from the dotted line. This phenomenon suggests that certain models may exhibit large prediction errors when dealing with extreme values, indicating that their robustness may need to be further optimised.

As can be seen from Table 3, the performance of the six models varies significantly, with Linear Regression exhibiting the weakest predictive capability compared to more sophisticated machine learning models such as SVR, AdaBoost, BP-NN, XGBoost, and GB.

(1) The R² values reveal substantial differences in the explanatory power of each model. SVR stands out with the highest R² (0.8382), indicating superior ability to capture the variance in effluent quality and delivering the most accurate fit overall. XGBoost follows closely (0.8131), outperforming other ensemble methods and highlighting its strong generalization capacity. BP-NN (0.8030), AdaBoost (0.7979), and GB (0.7916) exhibit moderately strong performance but with slightly reduced fidelity in modeling complex nonlinear relationships. In contrast, Linear Regression lags significantly behind with the lowest R² (0.7358), underscoring its inadequacy in addressing the multifactorial and nonlinear nature of effluent quality prediction.
(2) The Mean Absolute Percentage Error (MAPE) provides additional insight into predictive accuracy across models. XGBoost once again demonstrates superior performance with the lowest MAPE (6.1108%), reinforcing its advantage in minimizing relative prediction errors. In contrast, BP-NN (6.9562%) and especially Linear Regression (8.6149%) show noticeably higher MAPE values, indicating reduced reliability in capturing actual effluent quality trends. Meanwhile, the Mean Bias Error (MBE) offers a complementary perspective on prediction deviation. SVR (0.1124) and XGBoost (0.2459) yield the smallest biases, suggesting well-balanced predictions close to observed values. In comparison, BP-NN (−0.6542) and AdaBoost (−0.6268) exhibit more pronounced negative biases, reflecting a tendency to systematically underpredict effluent quality.
(3) The standard deviation of MAPE (MAPE Std Dev) offers valuable insights into the stability and consistency of model predictions. BP-NN (1.1719) and SVR (1.0624) exhibit the highest variability, indicating that their predictive performance is less consistent across different test samples. In contrast, XGBoost (0.7830) and AdaBoost (0.7721) display lower prediction fluctuation, suggesting more stable and reliable outputs under varying conditions.

Further reinforcing these findings, the 95% confidence intervals (CI) for MAPE reveal distinct differences in prediction uncertainty. Linear Regression (6.8429%–10.6754%) not only shows the widest error range but also confirms its lack of precision and robustness. In stark contrast, XGBoost (4.6054%–7.6602%) achieves the narrowest CI, reflecting both high accuracy and high confidence in its predictions. Although SVR (4.8061%–8.8652%) reports relatively low average error, its wider CI suggests less stable generalization performance compared to XGBoost.

(4) To assess the robustness of model predictions, paired t-tests and Wilcoxon signed-rank tests were conducted. The paired t-test p-values suggest that there is no statistically significant difference between the predicted and actual values for most models, with all p-values greater than 0.05. Similarly, the Wilcoxon test p-values indicate no significant difference in the distribution of errors, reinforcing the reliability of the models’ predictions.

Linear Regression performs significantly worse than the machine learning models across all metrics. Its higher MAPE (8.6149%), lower R² (0.7358), and highest absolute error range that it lacks the predictive power and stability required for accurate effluent quality prediction. These results confirm that the more complex models in this study not only provide superior predictive power but also exhibit greater stability, justifying their preference over simpler linear models for this task.

(5) In addition to prediction accuracy and robustness, training time is also a critical factor when evaluating model practicality, particularly for real-time or resource-constrained applications. As shown in Table 3, the training time varied significantly among the six models. AdaBoost and BP-NN required the longest training durations (10.18 s and 5.04 s, respectively), mainly due to iterative ensemble learning and multi-layer backpropagation. In contrast, Support Vector Regression (SVR), XGBoost, and Gradient Boosting (GB) achieved a balance between computational efficiency and predictive accuracy, with training times of 1.27 s, 2.84 s, and 1.97 s, respectively. Linear Regression demonstrated the fastest training time (0.01 s), but at the cost of significantly lower predictive performance.

#### 3.2.2 Residual analysis and error distribution.

Residual analysis is a fundamental approach to assessing model performance by examining the differences between actual and predicted values. An ideal model should exhibit residuals symmetrically distributed around zero, with a controlled spread and minimal outliers, ensuring balanced generalization across both training and testing datasets. To evaluate the predictive reliability of different models, the residual distributions of AdaBoost, BP-NN, SVR, XGBoost, and GB are analyzed using boxplots.As can be seen from Fig 3.

The residuals of AdaBoost demonstrate a relatively symmetric distribution around zero, particularly in the training dataset. However, the test residuals exhibit a slight shift, indicating some degree of bias in prediction errors. The residual spread is more compact in the training set but expands in the test set, suggesting a reduction in predictive consistency when applied to unseen data. No outliers are observed in either dataset, indicating a stable prediction across most samples. The generalization capability is moderate, but the increased variance in test residuals suggests a slight decline in predictive robustness.

The residual distribution of BP-NN appears moderately symmetric, though a slight skewness is noticeable in the test set, indicating some bias in predictions. The residuals are widely spread in both training and test datasets, reflecting higher variance and suggesting that BP-NN predictions fluctuate significantly. Outliers are present in the test dataset, implying that certain samples are particularly challenging for the model. The generalization ability of BP-NN is less stable, as the spread of residuals and the presence of outliers indicate potential difficulties in maintaining prediction consistency across datasets.

The residuals of SVR are nearly symmetric around zero, particularly in the training set, but slight asymmetry is observed in the test set. The residual spread is moderate in both datasets, though the test residuals exhibit a larger variance, suggesting that SVR struggles to maintain consistency on unseen samples. A single outlier is observed in the test dataset. The overall generalization capability is reasonable, but the increased variability in the test residuals suggests that SVR may be influenced by specific sample variations.

XGBoost residuals exhibit a relatively symmetric distribution around zero in both training and test datasets, indicating low prediction bias. The residual spread remains well-controlled, with moderate variability that is consistent across datasets. No outliers are detected, suggesting that XGBoost maintains stable performance across different samples. The strong alignment of residual distributions between training and testing datasets highlights its effective generalization capability, ensuring robust performance on unseen data.

The residuals of GB are highly symmetric around zero in both training and test datasets, demonstrating balanced prediction errors. The residual spread in the training set is notably compact, suggesting a strong fit, while the test residuals show slightly greater variability, though still within a controlled range. No outliers are present in either dataset, further supporting the model’s stability. The consistency of residual distributions across datasets indicates strong generalization performance.

#### 3.2.3 Model convergence analysis.

As can be seen from Fig 4, model convergence analysis is essential for evaluating the stability and efficiency of machine learning models. A well-converging model indicates that it has effectively learned from the data and has reached an optimal balance between bias and variance. Poor convergence behavior, on the other hand, may result in suboptimal performance or overfitting. In this study, the convergence behavior of five models—Adaboost, BP-NN,SVR, XGBoost and GB—was analyzed by plotting the MSE against the number of iterations or boosting steps. The results of these convergence curves provide insights into how efficiently each model minimizes the error and reaches stability.

Examining the final results of the models, all five algorithms successfully achieved convergence, with their MSE stabilizing at the later stages of training. BP-NN achieved the lowest MSE after approximately 60 epochs, demonstrating its ability to capture nonlinear relationships effectively. SVR reached a stable minimum MSE within 20 iterations, suggesting that the model converges quickly given an optimal choice of hyperparameters. GB and XGBoost both exhibited effective learning, with their MSEs decreasing steadily and reaching stable values.Adaboost, while achieving convergence, demonstrated a slower decline in MSE, with the error decreasing gradually over more iterations. This suggests that Adaboost requires a larger number of estimators to reach an optimal performance level, aligning with its iterative nature of updating weak learners.

Comparing the convergence speed and trends, BP-NN and SVR displayed the fastest convergence. BP-NN’s convergence curve initially fluctuated significantly due to weight adjustments but stabilized after around 30 epochs. SVR, leveraging the kernel trick, exhibited rapid error reduction, reaching convergence in fewer than 20 iterations. Adaboost, in contrast, had a steadier but slower convergence rate, with the MSE continuing to decrease even after 100 iterations, though at a diminishing rate. Both GB and XGBoost showed a steady reduction in MSE, indicating effective learning with boosting approaches.

Overall, BP-NN and SVR demonstrated superior convergence efficiency, achieving stability in fewer iterations. Boosting models, while taking more iterations to converge, maintained stable learning trends.

## 4 Discussion and practical application scenarios

### 4.1 Discussion

This study evaluated the performance of five supervised learning models—SVR, XGBoost, GB, AdaBoost, and BP-NN—for predicting effluent quality in wastewater treatment plants (WWTPs). The models were assessed across multiple dimensions, including fitting accuracy (R²), error control, generalization ability, convergence stability, and statistical robustness. Based on the comparative analysis of these metrics and their observed behavior in practical application scenarios, a comprehensive discussion is presented below to highlight each model’s strengths, limitations, and suitability for real-world implementation.

Among the five models, SVR achieved the highest R², indicating the strongest fitting capability to the training dataset. This can be attributed to SVR’s strategy of optimizing a separating hyperplane in high-dimensional space, which enables it to perform well on small datasets. However, its performance in relative error and bias control was suboptimal, as evidenced by a higher variance in test set predictions and slightly asymmetric residuals. These patterns suggest that SVR’s sensitivity to global data distributions is limited, which compromises its generalization ability. Furthermore, while SVR converged rapidly, its predictive stability remained relatively low, making it prone to larger deviations when handling unseen or outlier samples. Given the fluctuating and multifactorial nature of effluent quality in real WWTP operations, SVR’s reliance on local optimization makes it less suitable for large-scale, multivariable, or long-term predictive tasks.

XGBoost, while slightly behind SVR in R², outperformed all other models in error control, generalization, and convergence stability. This superiority stems from its use of both L1 and L2 regularization terms and the stepwise optimization mechanism of gradient boosting trees, which collectively enhance robustness and mitigate overfitting. The residuals of XGBoost were symmetrically distributed with low variance across training and test sets, indicating balanced prediction behavior and strong generalization. Although XGBoost required more iterations to reach convergence, it consistently demonstrated stable learning trends and minimized prediction errors, making it highly suitable for precision-focused, operational wastewater monitoring tasks, where pollutant concentrations are subject to frequent and irregular fluctuations.

GB exhibited performance comparable to XGBoost in terms of convergence pattern and residual symmetry, but with slightly lower R² and higher prediction error variance. The lack of regularization mechanisms and parallel computation optimizations in traditional GB may explain its weaker generalization capacity, particularly when applied to small or noisy datasets. While GB remains a viable alternative to XGBoost, especially in scenarios where model interpretability or implementation simplicity is prioritized, its higher sensitivity to data noise could limit reliability under conditions of high variability in pollutant concentrations.

AdaBoost showed the weakest R² among the ensemble models, reflecting limited effectiveness in modeling complex nonlinear relationships. This limitation likely arises from AdaBoost’s iterative adjustment mechanism for weak learners, which is well-suited for linear or mildly nonlinear problems but less capable of capturing intricate dependencies in multivariate pollutant data. AdaBoost also converged more slowly than the other models, requiring a greater number of iterations to stabilize, and showed greater residual dispersion in the test set, all of which indicate suboptimal generalization performance. Nevertheless, AdaBoost may still be appropriate in computationally unrestricted environments where linear interpretability is valued more than predictive precision.

BP-NN outperformed AdaBoost and GB in R² but exhibited higher prediction errors and greater variance, indicating insufficient stability. This model’s performance is closely tied to training data volume; neural networks typically require large datasets to effectively capture nonlinear relationships. In this study, the dataset size may have been insufficient for BP-NN to learn the complex patterns in effluent quality variation. The convergence curve of BP-NN also fluctuated significantly in the early training phase, and outliers were detected in the test set, highlighting its limited robustness and lower reliability on smaller-scale data. However, in future studies involving larger, long-term datasets, BP-NN may yield improved performance and become a more competitive choice.

To verify the statistical reliability of these models, both paired T-tests and Wilcoxon signed-rank tests were conducted to compare the distributions of predicted and actual values. For all five machine learning models, the p-values exceeded 0.05 in both tests, indicating no statistically significant difference between predicted and true values. This outcome suggests that, despite varying performance levels in terms of accuracy and generalization, each model delivers stable and consistent predictions from a statistical standpoint.

In conclusion, XGBoost emerges as the most suitable model for wastewater treatment effluent quality prediction due to its balanced performance across all key metrics. Its superior error control, generalization, and robustness make it highly adaptable to the real-world complexities of WWTP operations. SVR, while offering excellent fitting performance, lacks the broader reliability needed for operational deployment. GB is a viable but less stable alternative to XGBoost. AdaBoost and BP-NN, given their constraints on nonlinearity handling and data requirements respectively, are less recommended under current data conditions but may still hold potential in specific expanded datasets or application contexts.

Compared to previous studies on wastewater effluent quality prediction, the models developed in this work exhibit competitive or improved performance. For instance, Chen et al. (2023) employed SVR and BP neural networks to predict COD concentrations, reporting an R² of 0.78 for SVR and up to 0.81 for BP neural networks following MPSO-based optimization [36]. In our study, without relying on metaheuristic tuning, the SVR model achieved an R² of 0.8382, while BP-NN and XGBoost yielded R² values of 0.8030 and 0.8131, respectively, for EQI prediction. These results are particularly notable given that EQI represents a composite water quality index encompassing multiple pollutant parameters, thereby posing a more complex prediction task than single-indicator modeling. The models were trained using standardized cross-validation protocols, and hyperparameters were optimized via GridSearchCV to ensure reproducibility and comparability. Collectively, these findings suggest that the selected algorithms—particularly SVR and XGBoost—are not only effective for single-parameter modeling, as shown in previous studies, but also robust in capturing integrated effluent characteristics in more demanding predictive contexts.

### 4.2 Practical application scenarios

This study demonstrates the performance of each of them in terms of prediction accuracy, error control, and model stability by comparing the application of multiple machine learning models for effluent water quality prediction in WWTP. Accurate water quality prediction is crucial for process regulation, risk management and resource optimisation in the daily operation of WWTP. Based on the findings of this paper, the following are possible application scenarios of these machine learning models in wastewater treatment management and the potential benefits they bring:

(1) Process optimisation and dynamic adjustment: the variation of the concentration of different pollutants during wastewater treatment has a direct impact on the setting of treatment process parameters. With the machine learning model in this study, managers can predict the trend of effluent water quality in real time, and then adjust the treatment process parameters, such as aeration intensity, sedimentation time, and the amount of pharmaceutical injection, before the pollutant concentration reaches a critical value. This data-driven dynamic adjustment mechanism can improve treatment efficiency and avoid exceeding the effluent water quality standard due to untimely process adjustment. At the same time, with the continuous optimisation of the model, the system can achieve synergistic regulation of a variety of treatment process parameters to achieve the best operating conditions.
(2) Construction of Intelligent Decision Support System: The successful application of multiple machine learning models in this study provides a technical basis for the development of an intelligent decision support system (DSS) based on prediction. The system can combine real-time monitoring data and prediction results to automatically generate operational recommendations to assist managers in making more accurate and timely decisions. For example, when the model predicts that the effluent water quality will deviate from the set standard, the system can recommend appropriate process adjustment strategies to reduce the response time of manual operations. In addition, the decision support system can also provide long-term optimisation recommendations, such as reducing energy consumption, reducing the use of chemicals, and ultimately improve the economic and environmental benefits of WWTP.
(3) Prediction and early warning and risk management: Risk management in the wastewater treatment process is a challenging problem, especially when there are large fluctuations in treatment flow or sudden increases in influent load. The prediction model in this study can identify potential water quality anomalies in advance and provide early warning signals. This feature helps WWTP to take preventive measures, such as activating standby equipment, increasing treatment capacity, or adjusting operating parameters in advance, to reduce the impact of emergencies on water quality. In addition, by analysing the performance of the model in different scenarios, it can provide managers with more targeted contingency plans, thus improving the robustness and safety of the system.
(4) Integration of automation and remote control system: The intelligent trend of wastewater treatment is gradually shifting from pure monitoring to automation control. The prediction model in this study can be integrated with the automation control system to achieve remote control and real-time regulation. Through the dynamic response of the automation system, the model prediction results can be used to trigger immediate process adjustment, reducing the frequency and error of human intervention. This integrated system not only significantly improves the operational efficiency of wastewater treatment, but also reduces manpower costs and improves the control accuracy of complex processes, promoting the overall intelligence of WWTP.
(5) Practical Implications for Real-Time WWTP Management and Sustainable Development Goal (SDG) 6: Sustainable Development Goal 6 (SDG 6) is part of the United Nations 2030 Agenda for Sustainable Development, aiming to “ensure availability and sustainable management of water and sanitation for all”. It emphasizes improving water quality, increasing water-use efficiency, expanding wastewater treatment coverage, and ensuring the protection of water-related ecosystems. Achieving this goal requires advancements in wastewater treatment technologies, predictive monitoring, and resource optimization, making machine learning a promising tool in supporting sustainable water management.In real-time WWTP management, machine learning-based predictive systems enable proactive decision-making, helping to minimize wastewater discharge violations and optimize resource consumption. By integrating real-time monitoring data with predictive analytics, WWTP can reduce unnecessary energy use, optimize chemical dosing, and lower greenhouse gas emissions, contributing to both economic savings and environmental sustainability. Furthermore, the ability of machine learning models to adapt to different operational conditions ensures more resilient wastewater treatment strategies, making WWTP better equipped to handle urban expansion, industrial growth, and climate-induced water challenges.

## 5 Outlook for future research

In this study, the prediction of effluent quality in wastewater treatment plants WWTP using machine learning models was explored in depth, demonstrating the potential of supervised learning techniques in wastewater management. However, several limitations remain, particularly regarding the dataset size and the generalization capability of the models. These limitations must be addressed in future research to further enhance the reliability and applicability of machine learning-based wastewater prediction.

One of the primary constraints of this study is the relatively small dataset, which consists of only 84 monthly records. While the models performed satisfactorily on this dataset, real-world WWTP operations involve more complex environmental factors and greater data variability. The limited dataset size may affect the robustness of the models when applied to different WWTP or varying climatic conditions. Future research should focus on expanding the dataset by incorporating larger-scale historical records, data collected under different climatic conditions, and high-frequency real-time monitoring data. By increasing data diversity, models can be trained to better adapt to dynamic operational conditions, including extreme weather events or emergency scenarios, thereby improving early warning capabilities and prediction accuracy.

To mitigate the limitations imposed by the dataset size, future studies may also explore data augmentation and enhancement techniques. Generative Adversarial Networks (GANs) could be used to generate synthetic data, increasing the coverage and variability of the dataset while maintaining realistic pollutant concentration distributions. Additionally, transfer learning techniques could be leveraged to utilize large-scale data from related domains, enhancing model performance even when available WWTP-specific data is limited. Furthermore, the integration of real-time sensor data from WWTP will support continuous monitoring, process optimization, and real-time prediction, making machine learning models more adaptable to practical applications.

At the algorithmic level, this study primarily focused on traditional machine learning models (e.g., SVR, AdaBoost, BP-NN, XGBoost, and GB), which have limitations in capturing complex time-series relationships. Future research should explore more advanced deep learning models, such as Long Short-Term Memory Networks (LSTM) and Convolutional Neural Networks (CNN), which are well-suited for processing time-series data and capturing intricate nonlinear relationships in wastewater treatment processes. Additionally, reinforcement learning (RL) techniques could be integrated with predictive models to create a closed-loop intelligent control system, optimizing operational parameters dynamically and advancing automation in WWTP management.Furthermore, hybrid approaches combining machine learning with multi-objective optimization frameworks are gaining increasing attention in wastewater management. These approaches not only aim to improve prediction accuracy but also seek to balance competing operational goals, such as minimizing effluent pollutant levels, energy consumption, chemical usage, and treatment costs. For example, models like XGBoost or LSTM can be embedded within evolutionary optimization frameworks (e.g., NSGA-II, MOEA/D) or metaheuristic algorithms (e.g., particle swarm optimization, genetic algorithms) to enable simultaneous optimization of multiple performance metrics under various constraints. These hybrid strategies are particularly valuable in real-world WWTP scenarios, where decision-making often involves complex trade-offs among efficiency, sustainability, and regulatory compliance.

Another limitation of this study is the independent evaluation of individual machine learning models, without exploring the potential benefits of hybrid approaches. Future research should consider integrating multiple models in ensemble learning frameworks, leveraging the complementary strengths of different algorithms to improve prediction accuracy and robustness. Moreover, multi-objective optimization approaches could be incorporated, allowing effluent quality prediction models to be combined with energy consumption optimization, cost reduction strategies, and environmental impact assessments. By balancing these objectives, WWTP can enhance operational efficiency while minimizing resource consumption and maximizing environmental benefits.

In summary, while this study provides valuable insights into the application of machine learning for wastewater treatment prediction, future research should focus on expanding dataset size, introducing advanced algorithms, integrating real-time monitoring data, and incorporating multi-objective optimization strategies. Addressing these limitations will unlock the full potential of machine learning in intelligent wastewater management, providing more accurate, reliable, and efficient decision-support systems for WWTP operations. Further exploration in these directions will contribute to the sustainable development of the wastewater treatment industry, reinforcing the role of data-driven intelligence in optimizing environmental management practices.
